# The case of a patient with MIRAGE syndrome with familial dysautonomia-like symptoms

**DOI:** 10.1038/s41439-021-00158-6

**Published:** 2021-07-12

**Authors:** Yuki Kawashima-Sonoyama, Keisuke Okuno, Tomotsune Dohmoto, Kanako Tanase-Nakao, Satoshi Narumi, Noriyuki Namba

**Affiliations:** 1 Division of Pediatrics & Perinatology, Tottori University Faculty of Medicine, Yonago, Japan; 2 Department of Pediatrics, Shimane University Faculty of Medicine, Izumo, Japan; 3 Department of Pediatrics, Tottori Prefectural Central Hospital, Tottori, Japan; 4 Department of Molecular Endocrinology, National Research Institute for Child Health and Development, Tokyo, Japan

**Keywords:** Disease genetics, Diseases

## Abstract

We describe a case of posthumously diagnosed MIRAGE syndrome (Myelodysplasia, Infection, Restriction of growth, Adrenal hypoplasia, Genital problems, and Enteropathy) in a girl with a new pathogenic *SAMD9* variant (p.F437S), who was initially considered to have familial dysautonomia (FD)-like disease due to increased levels of catecholamine metabolites. Functional analyses of F437S-SAMD9 were performed, showing characteristics of disease-causing variants. This new *SAMD9* variant (p.F437S) also causes MIRAGE syndrome.

Myelodysplasia, infection, restriction of growth, adrenal hypoplasia, genital problems, and enteropathy (MIRAGE) syndrome is a genetic disorder caused by gain-of-function *SAMD9* mutations, which lead to a multisystemic growth restriction disorder^1^. Recently, several cases of patients with MIRAGE syndrome presenting with dysautonomia have been reported^2–4^. Familial dysautonomia (FD) is a rare genetic disease caused by a founder variant in the *ELP1* gene^5–9^. FD involves impaired development of sensory and afferent autonomic nerves, which leads to widespread organ dysfunction and mortality^8,9^. Early signs and symptoms include hypotonia, feeding difficulties, growth restriction, a lack of tears, and frequent lung infections^9^. Most of these symptoms are also observed in patients with MIRAGE syndrome. In several patients with FD-like symptoms, no *ELP1* mutation was identified^10,11^. This study describes the case of a patient who presented with FD-like symptoms and myelodysplastic syndrome (MDS) with monosomy 7 and was posthumously diagnosed as having MIRAGE syndrome due to a rare *SAMD9* variant.

The patient was born with severe asphyxia at 37.3 weeks of gestation (birth weight: 2.002 g [−2.1 SD]; birth length: 44 cm [−1.6 SD]). The newborn had dysphagia, repeated apneic attacks, thrombocytopenia, and anemia. Additionally, dysautonomic conditions such as hyperhidrosis, paresthesia, and lacrimal deficiency were observed. External features included a flat upper lip and tongue mushroom papilla loss (Fig. 1A). The results of array comparative genomic hybridization were normal, and the patient was discharged at 4 months. At 8 months, the patient was diagnosed with MDS associated with monosomy 7. At 16 months, the patient experienced irritable contractions after the methacholine eye drop test, with increased levels of catecholamine metabolites (plasma norepinephrine: 1.79 ng/mL [range: 0.15–0.57], urine homovanillic acid: 14.4 mg/g creatinine [range: 2.1–8.2], urine vanillylmandelic acid: 2.93 mg/g creatinine [range: 2.4–8.3], ratio of homovanillic acid to vanillylmandelic acid: 2.93 [range < 1.5])^12^ and a negative histamine intradermal reaction (Fig. 1B). On the basis of these findings, we suspected FD. However, the analysis of the *ELP1* gene revealed no mutations; therefore, the patient was considered to have an FD-like disease. Growth retardation continued (Fig. 1C). The patient experienced intermittent pneumonia and vomiting episodes with colonic dilation despite undergoing Nissen surgery and gastrostomy at 17 months of age. There was no adrenal insufficiency. Mental retardation was noted at 32 months. At 5.5 years, with progressing pancytopenia, bone marrow tests showed the progression of MDS to refractory anemia, with excess type 2 blasts (RAEB-2). Allogeneic bone marrow transplantation was performed at 5.75 years. Despite achieving complete remission, RAEB-2 recurred at 6.33 years, leading to the patient’s death.

**Fig. 1:**
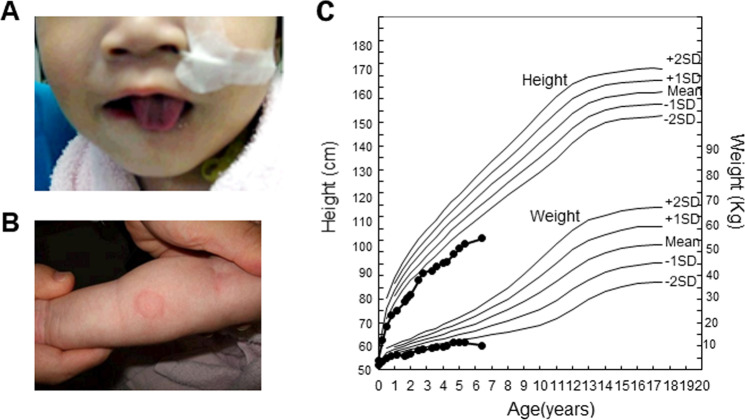
Clinical findings. **A** The patient at 16 months of age, showing the flat upper lip and loss of tongue mushroom papillae. **B** Image 15 min after the histamine intradermal reaction test. Swelling and flare were not identified. **C** Patient cell growth curve.

The genetic profile of the patient remained unclear even after her death in 2013. MIRAGE syndrome was suspected when the condition was first reported in 2016. Genomic DNA and RNA were extracted from leukocytes, saliva, and fibroblasts obtained from the patient’s right anterior chest skin. *ELP1* expression was evaluated, and *SAMD9* was sequenced from genomic DNA isolated from fibroblasts, as previously described^3,13^. Genomic analysis revealed a heterozygous de novo missense *SAMD9* mutation (NM_017654.4: c.1310T>C, p.F437S) (Fig. 2A). This mutation has not been reported in the gnomAD database, the 1000 Genomes database, or the Human Gene Mutation Database. The phenylalanine at position 437 (F437) is conserved across several species (Fig. 2B).

**Fig. 2:**
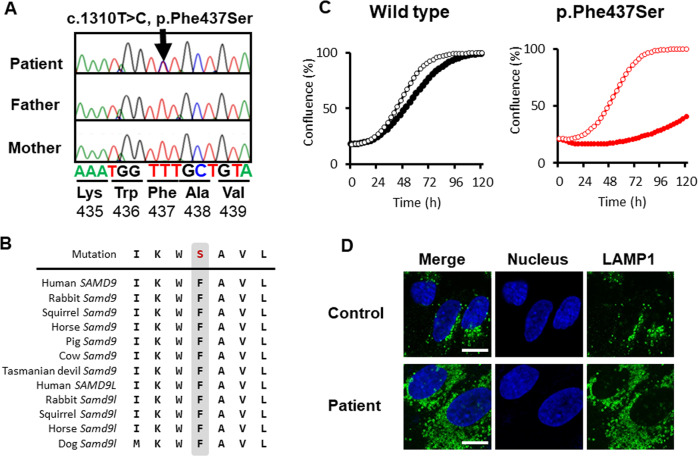
Analysis of the *SAMD9* variant in the patient. **A** Patient and parent polymerase chain reaction-amplified *SAMD9* electropherograms; a heterozygous mutation was identified in the patient (arrow). **B** Alignment (Clustal software) showing the conserved F437 residue. **C** Growth curves of HEK293 cells transfected without (empty circles) or with (solid circles) wild-type (WT)-SAMD9 (left panel) and F437S-SAMD9 (right panel) plasmids. **D** Confocal image of patient-derived fibroblasts showing lysosomes and nuclei stained with anti-LAMP-1 antibody (green) and DAPI (blue), respectively.

The effect of the F437S mutation on cell growth was evaluated in HEK293 cells expressing the native or mutated SAMD9 protein [Wild type (WT) and F437S, respectively] upon induction, which were established and analyzed to measure the growth rate, as previously described^3^. WT-SAMD9 expression induced mild growth suppression, as previously reported, whereas F437S-SAMD9 inhibited cell growth (Fig. 2C). LAMP-1 visualization showed a remarkable increase in the lysosome number in the patient’s fibroblasts (Fig. 2D), which is a characteristic of MIRAGE syndrome^1,4^. Thus, the patient was posthumously diagnosed with this syndrome.

Written informed consent for genetic analysis and the use of patient photos for publication was obtained from the patient’s parents. This study was approved by the Ethical Review Board of the Tottori University School of Medicine, Japan (No. 19A066G), and conducted according to the principles of the Declaration of Helsinki.

Dysautonomia, including insensitivity and anhidrosis, has been reported in several MIRAGE syndrome cases^2–4^. Conversely, dysautonomia at birth, feeding difficulties, poor growth, and frequent lung infections are observed only in FD and not in any other form of dysautonomia^9^. Interestingly, our patient also tested positive for laboratory test results common to FD, such as irritable contractions with the methacholine eye drop test, increased catecholamine metabolite levels, and a negative histamine intradermal reaction^9,12^. Further studies are warranted to determine whether *ELP1* interacts with *SAMD9*. In conclusion, the new *SAMD9* variant (p.F437S) also causes MIRAGE syndrome, and dysautonomia is considered a symptom that indicates the possibility of MIRAGE syndrome.

## HGV database

The relevant data from this Data Report are hosted at the Human Genome Variation Database at 10.6084/m9.figshare.hgv.3042.
